# Post-fracture serum cytokine levels are not associated with a later diagnosis of complex regional pain syndrome: a case-control study nested in a prospective cohort study

**DOI:** 10.1186/s12883-022-02910-z

**Published:** 2022-10-12

**Authors:** Luke Parkitny, James H McAuley, Robert D. Herbert, Flavia Di Pietro, Aidan G Cashin, Michael C Ferraro, G. Lorimer Moseley

**Affiliations:** 1 Departments of Pediatrics-Neurology, Baylor College of Medicine, Houston, TX USA; 2 Jan and Dan Duncan Neurological Research Institute at Texas Children’s Hospital, Houston, TX USA; 3 Centre for Pain IMPACT Neuroscience Research Australia, University of New South Wales, Sydney, Australia; 4 School of Health Sciences, Faculty of Medicine, University of New South Wales, Sydney, Australia; 5 Curtin Medical School, Curtin University, Bentley Campus, Bentley, Australia; 6 Curtin Health Innovation Centre (CHIRI), Curtin University, Bentley, Australia; 7 University of South Australia, Adelaide, Australia

**Keywords:** Complex regional pain syndrome, Cytokines, Inflammation

## Abstract

**Background:**

Complex Regional Pain Syndrome (CRPS) is a disabling pain disorder that is most common after a distal limb fracture. While the acute systemic immune response to the injury is thought to play a role in the development of CRPS, this hypothesis has never been tested directly. Thus, we evaluated whether elevated levels of circulating pro-inflammatory cytokines early after a fracture were associated with the development of CRPS.

**Methods:**

We conducted a case-control study nested within a prospective cohort study. Individuals with wrist and/or hand fractures were recruited from specialist hand units. Baseline clinical data were obtained from participants within 28 days of fracture. CRPS status was determined 16 weeks after the fracture using a two-stage diagnostic process. Cytokine assays were obtained from all cases (defined using the Budapest criteria) and a random sample of those who did not have CRPS at 16 weeks. We calculated odds ratios with 95% confidence intervals to determine the risk of CRPS associated with the expression of each of 25 cytokines.

**Results:**

Baseline data were collected for 702 consenting participants, of whom 535 provided blood samples. Follow-up at 16 weeks was 97.2%. 15 (2.2% of the cohort) met the Budapest CRPS criteria and 69 (including those who met the Budapest criteria; 9.8%) met the International Association for the Study of Pain (IASP) CRPS criteria. In all of the primary analyses (using Budapest criteria) and 49/50 secondary analyses (using IASP criteria), 95% confidence intervals for the association between cytokine levels and the risk of subsequently developing CRPS included the null value (OR = 1). However, the confidence intervals were wide.

**Conclusion:**

There was no evidence that early post-injury expression of systemic cytokines was associated with a CRPS diagnosis 16 weeks after injury. This study does not provide support for the hypothesis that innate immune activation has a determinative role in the development of CRPS.

**Supplementary Information:**

The online version contains supplementary material available at 10.1186/s12883-022-02910-z.

## Background

It has been estimated that approximately 3–7% of people with a distal limb fracture develop Complex Regional Pain Syndrome (CRPS) [1, 2]. Although fracture is the most common trigger of CRPS [3], other injuries such as intravenous cannulation, arthroscopy, sprain, and blunt trauma have also been reported. The clinical course of CRPS is often protracted [1] and involves the development of pain and complex sensory, motor, cognitive, autonomic, neuropsychological and trophic changes, often resulting in substantial disability and emotional distress [4–9].

The key pathophysiological mechanisms of CRPS include dysregulation of cortical sensorimotor, vasomotor, and inflammatory functions [4]. Observations that CRPS-affected body parts can appear inflamed have long suggested that aberrant immune processes play a key role in the etiology of CRPS [4, 10]. There is robust evidence of abnormal cytokine expression in the blood, blister fluid, and cerebrospinal fluid in individuals with established CRPS [11]. Some individuals may in fact carry a greater immune-related risk for CRPS development, as the expression of class I and II human leukocyte antigens (HLA) has been reported as a risk factor for the disorder [12–16].

Three longitudinal studies have confirmed that the severity of early symptoms after a distal fracture is associated with the risk of developing CRPS. Two of these studies, with a combined sample size of 2145 fracture patients and a 4–7% incidence proportion of CRPS, found that moderate to severe acute pain intensity was associated with the later development of CRPS [1, 2]. Beerthuizen and colleagues reported that individuals who were later diagnosed with CRPS reported higher acute pain intensity (median 5.6, IQR (4, 7)) than people who did not develop CRPS (median 3.2, IQR (1, 5)) (1). Similarly, Moseley and colleagues found that people reporting two-day average acute pain intensity of ≥ 5 out of 10 were at greater risk of later developing CRPS (likelihood ratio for a 3–4 pain rating was 0.89, 95% CI (2.9, 2.72); 5–6 pain was 15.1, 95% CI (10.6, 21.4); and 7–8 pain was 78.9, 95% CI (35, 178)) [2]. In the third study, by Goris et al. [17], participants were assigned a regional inflammatory score based on pain intensity, skin temperature differences, color, edema, and range of motion. Participants with a high regional inflammatory score at baseline were more likely to experience a protracted recovery (r^2^ = 0.92, p = 0.01) but these findings were not specific to CRPS. This study also found that inter-limb difference in venous oxygenation was not predictive of CRPS. While none of these studies attempted to quantify immune activity directly, they suggest that early post-injury events such as inflammation may affect pain and recovery, and pose a risk for the development of CRPS.

To our knowledge, no study has directly tested whether systemic immune activation after injury has a role in the development of CRPS. Thus, the primary aim of this study was to test whether the post-injury systemic cytokine profile measured within 28 days of a fracture influences the subsequent development of CRPS.

## Methods

This study was conducted in accordance with the Helsinki Declaration and received human research ethics approval from South Eastern Sydney and Illawarra Health Service (HREC ref 10/051). All participants provided written informed consent prior to participation. We report the study in accordance with the Strengthening The Reporting of Observational Studies in Epidemiology (STROBE) statement for case-control studies (Additional file 1) [18].

### Participants

The design was a case-control study nested in a prospective cohort study. We recruited eligible individuals attending specialist hand units at three public hospitals in Sydney, Australia. The cohort included participants aged 18–75 who presented to a Sydney metropolitan fracture clinic within 28 days of a clinically confirmed unilateral fracture of the distal third of the radius, ulna, carpal bones, or metacarpals, and who were sufficiently proficient in the English language to enable study participation. We excluded individuals who had a diagnosis of CRPS, a co-existing neurological illness, pathological fracture (e.g. related to malignancy), were pregnant, or had any coexisting illness that the treating surgeon felt would markedly alter normal treatment.

### Study procedures

Baseline data were obtained within 28 days of the fracture. Where possible this took place during the individual’s first outpatient hospital visit. Baseline data included medical history and self-report of clinical variables. In addition, blood samples were collected. 16 weeks after the fracture, all individuals were followed up by telephone and, if there was any indication of CRPS, an in-person clinical interview was conducted to determine if the individual had CRPS. Funding was not available to test all blood samples collected at baseline so, after all baseline and outcome data were collected, exposure data were collected (assays were conducted of cytokine levels in blood samples) only for cases and controls, not for the entire cohort. The resulting nested case-control data were used to examine associations between exposure and the development of CRPS.

### Baseline assessment

Self-reported average severity of hand or wrist pain over the preceding 48 h was assessed using an 11-point numerical rating scale (NRS) [19–22]. For purposes of cohort characterization, we collected data on patient psychological and functional states. Depression, anxiety, and stress were assessed using the 21-item Depression, Anxiety, and Stress Scale (DASS-21) [23–28]. Participants also completed the short version of the Disabilities of the Arm, Shoulder and Hand (quickDASH) questionnaire [29, 30].

### Identification of cases and selection of controls

The primary outcome of the study was development of CRPS within 16 weeks of fracture. A two-stage process was used to diagnose CRPS. First, at the 16-week follow-up, study participants were interviewed by telephone using a set of standardized questions to assess the symptoms or signs of CRPS based on the Budapest [31] and IASP [32] diagnostic criteria (Appendix 1 in Additional file 2). Individuals who reported ongoing pain and the presence of two or more signs or symptoms of CRPS were invited to attend an in-person clinical examination. The diagnostic examination was conducted by investigator JHM who was blinded to the initial clinical details of the individual and baseline pain score (Appendix 2 in Additional file 2). A second blinded investigator, GLM, a CRPS expert, made a diagnosis of CRPS after examining these data.

The primary analyses were conducted using the Budapest criteria for CRPS. To meet the Budapest criteria, individuals were required to report the presence of continuing pain, have symptoms in at least three of four categories (sensory, vasomotor, sudomotor/edema, motor/and trophic), and present with at least two signs in these same categories. Individuals could only be assigned to the CRPS (Budapest) group after a clinical examination. We did not discriminate between CRPS-I and CRPS-II, which are diagnosed based on the absence or presence, respectively, of an associated nerve injury.

Additional analyses were conducted using the IASP criteria for CRPS. To meet the IASP criteria, individuals were required, during the 16-week telephone interview, to report continuing pain, allodynia, or hyperalgesia; and report the presence of edema, skin blood flow changes, or abnormal sudomotor activity in the region of pain at some time since the injury [32]. Individuals could be assigned to the CRPS (IASP) group with or without an in-person clinical examination. All of the individuals who met the Budapest criteria also met the IASP criteria.

An exclusive sampling strategy [33, 34] was used to randomly select controls from the members of the cohort who gave blood, were followed up and were not cases at the follow-up assessment.

### Ascertainment of exposure

Serum cytokines were measured in blood samples that were collected at baseline and prepared and stored using standardized protocols for < 2 years prior to analysis to minimize cytokine degradation [35–38]. Blood was collected from a vein in the cubital fossa of the uninjured arm into one BD Vacutainer serum separator tube. The sample was allowed to coagulate for 30–60 min at room temperature, then centrifuged at 1500 g for 15 min and stored on wet ice. Within 6 h of collection, the supernatant was aliquoted and frozen at − 80 °C.

Cytokine concentrations were measured using human 25-plex cytokine assay panels (Life Technologies, Carlsbad, California, U.S.A.) and Luminex technology. The standard manufacturer’s assay protocols were used except that we extended the standard curve by one dilution to improve the test sensitivity because we predicted low cytokine concentrations in the study. In short, test samples were incubated with the bead mixture at room temperature, then incubated with the biotinylated detection antibody mix, incubated with streptavidin-PE, and finally resuspended in an assay buffer for reading on a Bio-Rad reader using Bio-Plex Manager v5.0 to determine cytokine concentrations. To test assay accuracy, we determined intra-assay coefficients of variation (CV) for identical sample duplicates (Table 1). To minimize bias, in all analyses, measurements of serum cytokine concentrations that were below the lower limit of quantification (LLOQ) were replaced with values equal to half the LLOQ for the cytokine [39–41]. Cytokine concentrations above the upper limit of quantification (ULOQ) were replaced with values equal to the ULOQ.

**Table 1:** Results of primary outcomes analysis showing estimated odds ratios for the association between (for each cytokine) exposure and the risk of CRPS, where exposure is defined as being above the 80th centile for controls. Intra-assay coefficients of variation (CV) are provided for each cytokine. OR = Odds ratio; CI = confidence interval; IL-1β = interleukin-1 beta; IL-10 = interleukin-10; IFN-α = interferon alpha; IL-6 = interleukin-6; IL-12 = interleukin-12; RANTES = Regulated on Activation, Normal T Cell Expressed and Secreted; IL-13 = interleukin-13; IL-15 = interleukin-15; IL-17 = interleukin-17; MIP-1α = macrophage inflammatory protein-1 alpha; GM-CSF = granulocyte-macrophage colony stimulating-factor; MIP-1β = macrophage inflammatory protein-1 alpha; MCP-1 = monocyte chemoattractant protein-1; IL-5 = interleukin-5; IFN-γ = interferon gamma; TNF-α = tumor necrosis factor alpha; IL-1Ra = interleukin 1 receptor antagonist; IL-2 = interleukin-2; IL-7 = interleukin-7; IP-10 = interferon gamma-induced protein-10; IL-2r = interleukin-2 receptor; MIG = monokine induced by interferon-gamma; IL-4 = interleukin-4; IL-8 = interleukin-8.

Cytokine	CRPS Diagnosis OR	95% CI lower	95% CI upper	p	CV
IL-1β	0.81	0.08	3.90	0.78	5.4
IL-10	.	.	2.77	0.24	7.7
IFN-α	0.87	0.09	4.22	0.86	7.2
IL-6	0.37	0.01	2.62	0.32	5.7
IL-12	0.41	0.01	2.94	0.38	6.5
RANTES	2.01	0.43	7.72	0.26	6.2
eotaxin-1	0.81	0.08	3.93	0.79	6.1
IL-13	0.76	0.02	5.50	0.79	9.2
IL-15	0.37	0.01	2.60	0.32	5.6
IL-17	.	.	24.88	0.68	5.6
MIP-1α	0.81	0.08	3.93	0.79	6.7
GM-CSF	4.97	0.48	26.65	0.03	5.0
MIP-1β	0.80	0.08	3.89	0.78	4.7
MCP-1	0.36	0.01	2.58	0.32	5.8
IL-5	.	.	13.40	0.58	7.0
IFN-γ	3.24	0.07	26.66	0.26	5.2
TNF-α	1.22	0.13	5.98	0.80	5.9
IL-1Ra	0.36	0.01	2.59	0.32	5.5
IL-2	1.70	0.28	7.33	0.43	5.0
IL-7	1.02	0.11	5.11	0.98	6.0
IP-10	1.35	0.23	5.59	0.66	5.3
IL-2r	0.37	0.01	2.65	0.33	5.3
MIG	1.36	0.23	5.64	0.65	5.4
IL-4	1.09	0.02	8.00	0.94	5.8
IL-8	0.37	0.01	2.63	0.33	5.2

### Statistical analysis

Log binomial regression was used to quantify the univariate associations (RRs) between baseline pain intensity, upper limb disability, depression, anxiety, stress, and intraarticular fracture with subsequent development of CRPS.

The primary analysis quantified associations between cytokine levels and a diagnosis of CRPS made with the Budapest criteria. Participants were considered to have a high cytokine concentration (i.e. to be exposed) if the cytokine level was above the 80th centile for controls. We calculated odds ratios with exact confidence intervals for the association between each of the 25 cytokines and CRPS.

To test the robustness of the primary analysis, we conducted two secondary analyses. First, we used logistic regression to further explore the relationship between cytokine concentrations and CRPS, this time treating the log_10_ cytokine concentrations as continuous exposure variables. Then, because of the low frequency of CRPS (Budapest) in our cohort, we made a *post hoc* decision to repeat the primary analyses, this time defining CRPS cases less strictly. The cases in these analyses were individuals who satisfied the CRPS (IASP) criteria, of which a subset were those individuals who satisfied the CRPS (Budapest) criteria. In these analyses we adjusted for possible confounding by age (years), gender (male or female), and day since the injury. All analyses were conducted on Stata version 17.0 [42].

## Results

**Fig. 1:**
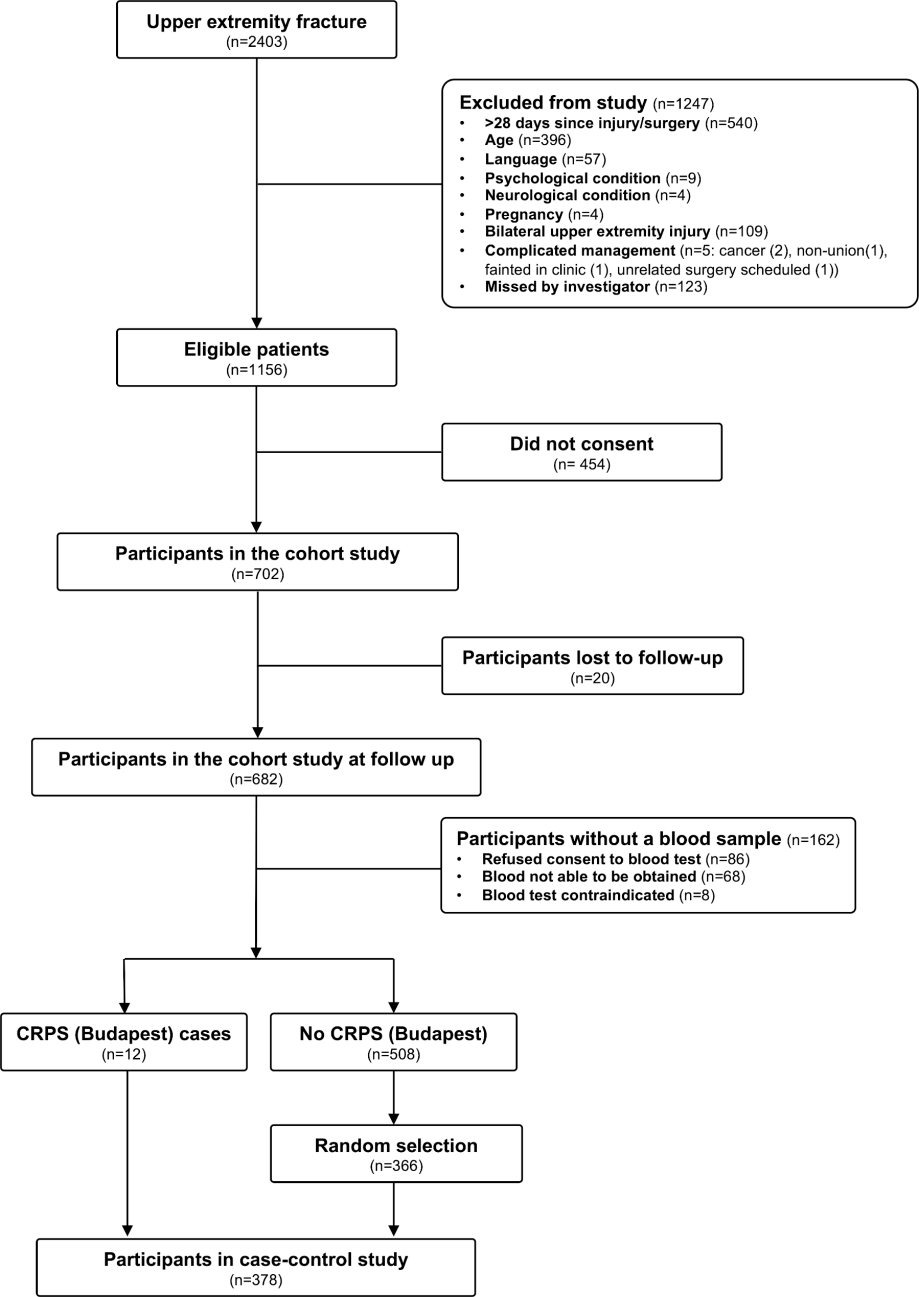
Recruitment flowchart showing numbers of included and excluded individuals at each stage of the study

Between August 2010 and March 2014 we screened 2403 consecutive individuals against the study inclusion/exclusion criteria. A study flowchart is presented in Fig. 1. 702 people were included in the study cohort. Follow-up data were available for 682 participants (97.1% follow-up). Of the whole cohort, 15 participants met the CRPS (Budapest) diagnostic criteria (risk = 2.4%, 95% CI 1.3–3.6%) and 69 (including the 15 who met the Budapest criteria) met the CRPS (IASP) diagnostic criteria (risk = 9.8%, 95% CI 7.8–12.3%) 16 weeks after fracture.

There were quite strong crude associations between baseline measures and the subsequent development of CRPS (Budapest). The relative risk (95% CI) was 4.0 (1.4–11.5) for fractures with articular involvement, 1.4 (1.2–1.8) for each unit on the 10-point NRS pain scale, 1.05 (1.01–1.08) for each unit on the 100-point quickDASH upper limb disability scale, 1.12 (1.04–1.21) for each unit on the 21-point DASS depression scale, 1.13 (1.04–1.23) for each unit on the 21-point DASS anxiety scale, and 1.12 (1.03–1.22) for each unit on the 21-point DASS.

The primary analyses included 12 individuals with CRPS (Budapest) who provided blood and 366 controls. Characteristics of these participants are shown in Table 2.

**Table 2:** Clinical and demographic characteristics of the study individuals. The first set of columns shows data from the whole cohort (N = 702), separated into those who gave blood and those who did not. p values are from independent samples t-tests for continuous variables and chi-square tests for categorical data. The second set of columns show data from case-control participants. Percentages that do not sum to 100% indicate missing data. CRPS (IASP) only participants are those who satisfied the IASP criteria for CRPS but not the Budapest criteria for CRPS. ^*^These individuals were conservatively managed at the time of assessment but subsequently were managed surgically. NRS = numerical rating scale; DASH = Disabilities of the Arm, Shoulder and Hand; DASS = Depression Anxiety Stress Scales

		Cohort Data			Case-control data			
		**Blood obtained**	**Blood not obtained**	**p**	**no CRPS**	**CRPS** **(IASP only)**	**CRPS (Budapest)**	**p**
Number		535	167		335	31	12	
Gender	female	173 (32.3%)	63 (37.7%)	0.20	108 (32.2%)	17 (54.8%)	7 (58.3%)	0.009
	male	362 (67.7%)	104 (62.3%)		227 (67.8%)	14 (45.2%)	5 (41.7%)	
Age, mean (SD)		38.6 (16.4)	38.5 (16.7)	0.97	38.1 (16.3)	51.0 (16.2)	37.8 (11.2)	< 0.001
Dominant hand	left	64 (12.0%)	19 (11.4%)	0.84	36 (10.7%)	2 (6.5%)	1 (8.3%)	0.73
	right	468 (87.5%)	147 (88.0%)		299 (89.3%)	29 (93.5%)	11 (91.7%)	
	missing	3 (0.6%)	1 (0.6%)					
Injured side	left	241 (45.0%)	69 (41.3%)	0.39	146 (43.6%)	14 (45.2%)	5 (41.7%)	0.98
	right	293 (54.8%)	98 (58.7%)		189 (56.4%)	17 (54.8%)	7 (58.3%)	
	missing	1 (0.2%)	0 (0.0%)					
Pain NRS, mean (SD)		2.8 (1.8)	2.9 (1.8)	0.38	2.7 (1.8)	3.7 (1.7)	4.4 (2.2)	< 0.001
QuickDASH score, mean (SD)		49.5 (15.9)	50.7 (17.2)	0.38	48.3 (15.7)	59.4 (16.3)	61.2 (18.1)	< 0.001
DASS21 Depression Score, mean (SD)		3.4 (4.0)	3.4 (4.7)	0.82	3.1 (3.9)	4.0 (4.0)	7.0 (4.2)	0.002
DASS21 Anxiety Score, mean (SD)		2.2 (3.0)	2.7 (3.9)	0.09	1.9 (2.7)	2.5 (3.7)	4.6 (4.7)	0.005
DASS21 Stress Score, mean (SD)		5.5 (4.6)	5.4 (5.1)	0.89	5.2 (4.5)	7.2 (4.4)	9.0 (4.7)	0.002
Recent or chronic illness	no	443 (82.8%)	132 (79.0%)	0.59	280 (83.6%)	25 (80.6%)	8 (66.7%)	0.3
	yes	92 (17.2%)	31 (18.6%)		55 (16.4%)	6 (19.4%)	4 (33.3%)	
	missing	0 (0.0%)	4 (2.4%)					
Asthma	no	483 (90.3%)	144 (86.2%)	0.47	303 (90.4%)	28 (90.3%)	10 (83.3%)	0.72
	yes	52 (9.7%)	19 (11.4%)		32 (9.6%)	3 (9.7%)	2 (16.7%)	
	missing	0 (0.0%)	4 (2.4%)					
Other pain	no	388 (72.5%)	119 (71.3%)	0.99	253 (75.5%)	19 (61.3%)	8 (66.7%)	0.19
	yes	147 (27.5%)	45 (26.9%)		82 (24.5%)	12 (38.7%)	4 (33.3%)	
	missing	0 (0.0%)	3 (1.8%)					
		**Cohort Data**			**Case-control data**			
		**Blood obtained**	**Blood not obtained**	**p**	**no CRPS**	**CRPS** **(IASP only)**	**CRPS (Budapest)**	**p**
Other inflammatory condition	no	497 (92.9%)	155 (92.8%)	0.32	308 (91.9%)	27 (87.1%)	12 (100.0%)	0.37
	yes	38 (7.1%)	8 (4.8%)		27 (8.1%)	4 (12.9%)	0 (0.0%)	
	missing	0 (0.0%)	4 (2.4%)					
Fracture site	distal radius	173 (32.3%)	50 (29.9%)	0.62	100 (29.9%)	15 (48.4%)	7 (58.3%)	0.48
	distal ulna	5 (0.9%)	1 (0.6%)		2 (0.6%)	0 (0.0%)	0 (0.0%)	
	metacarpal	206 (38.5%)	72 (43.1%)		135 (40.3%)	9 (29.0%)	2 (16.7%)	
	scaphoid	51 (9.5%)	18 (10.8%)		34 (10.1%)	3 (9.7%)	1 (8.3%)	
	other carpal bone	28 (5.2%)	4 (2.4%)		17 (5.1%)	1 (3.2%)	1 (8.3%)	
	multiple fractures	72 (13.5%)	22 (13.2%)		47 (14.0%)	3 (9.7%)	1 (8.3%)	
Intra-articular involvement	no	385 (72.0%)	115 (68.9%)	0.30	240 (71.6%)	25 (80.6%)	4 (33.3%)	0.011
	yes	122 (22.8%)	45 (26.9%)		80 (23.9%)	6 (19.4%)	7 (58.3%)	
	missing	28 (5.2%)	7 (4.2%)		15 (4.5%)	0 (0.0%)	1 (8.3%)	
Management	conservative	356 (66.5%)	110 (65.9%)	0.12	222 (66.3%)	19 (61.3%)	5 (41.7%)	0.16
	surgical	153 (28.6%)	42 (25.1%)		97 (29.0%)	8 (25.8%)	6 (50.0%)	
	surgical but seen preop.*	26 (4.9%)	15 (9.0%)		16 (4.8%)	4 (12.9%)	1 (8.3%)	
Days since fracture at baseline, mean (SD)		10.9 (5.0)	10.9 (5.3)	0.98	11.2 (5.3)	10.2 (4.8)	15.8 (6.2)	0.006

**Fig. 2:**
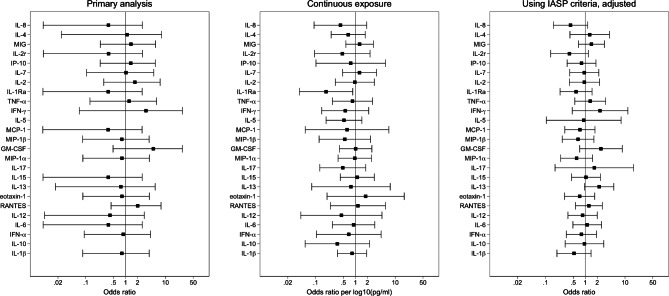
Results of analyses showing estimated odds ratios for the association between (for each cytokine) exposure and the risk of CRPS. Intra-assay coefficients of variation (CV) are provided for each cytokine. (a) primary analysis using the CRPS Budapest criteria, where exposure is defined as being above the 80th centile for controls; (b) secondary analysis treating log_10_ cytokine levels as continuous; (c) secondary analysis using the CRPS IASP criteria, where exposure is defined as being above the 80th centile for controls, adjusted for age (years), gender (male, female), and days since injury. Error bars represent the limits of the 95% confidence interval for the odds ratio. IL-8 = interleukin-8; IL-4 = interleukin-4; MIG = monokine induced by interferon-gamma; IL-2r = interleukin-2 receptor; IP-10 = interferon gamma-induced protein-10; IL-7 = interleukin-7; IL-2 = interleukin-2; IL-1Ra = interleukin 1 receptor antagonist; TNF-α = tumor necrosis factor alpha; IFN-γ = interferon gamma; IL-5 = interleukin-5; MCP-1 = monocyte chemoattractant protein-1; MIP-1β = macrophage inflammatory protein-1 beta; GM-CSF = granulocyte-macrophage colony stimulating-factor; MIP-1α = macrophage inflammatory protein-1 alpha; IL-17 = interleukin-17; IL-15 = interleukin-15; IL-13 = interleukin-13; RANTES = Regulated on Activation, Normal T Cell Expressed and Secreted; IL-12 = interleukin-12; IL-6 = interleukin-6; IFN-α = interferon alpha; IL-10 = interleukin-10; IL-1β = interleukin-1 beta

There was no evidence, in the primary analyses, of an association between cytokine concentration and subsequent diagnosis of CRPS (Table 1; Fig. 2). The 95% confidence intervals for all 25 cytokines included the null (OR = 1). However, confidence intervals for all estimates were wide. To increase statistical precision, we conducted further analyses which treated the (log_10_) cytokine levels as a continuous variable (Fig. 2, eTable 1 in Additional file 2), and used the more lenient IASP criteria for CRPS (Fig. 2; Table  1 in Additional file 2). The latter analysis included sufficient cases to allow some adjustment for selected confounders. In all but one of these 50 secondary analyses, the 95% confidence intervals for the association between and the risk of subsequently developing CRPS included the null value (OR = 1). The confidence intervals for these analyses were narrower than for the primary analysis but still wide.

## Discussion

We tested the hypothesis that the acute post-injury systemic cytokine profile is associated with the later diagnosis of CRPS. Because many factors modulate cytokine expression after injury – such as activation of the local host response at the site of injury, the stress-response, bone-healing, and surgery [43, 44] – we hypothesized that systemic cytokines would most closely reflect the complex mechanisms involved in the immune response to a localized injury. We found no evidence to support the theory that systemically circulating cytokines influence the development of CRPS, regardless of how CRPS was defined.

Multiple studies have reported that local, central, and systemic pro-inflammatory cytokines are upregulated in individuals with established CRPS [11]. However, these studies do not explain whether this aberrant inflammatory response precedes CRPS or whether it arises later, once the condition has developed. To our knowledge, only one previous study tested the inflammatory hypothesis of CRPS [17]. That study found that an inflammatory score was predictive of slow recovery but not specifically of the development of CRPS. However, that study assessed patients eight to nine weeks after the inciting injury and did not conduct a comprehensive assessment of immune function. In the present study, we comprehensively assessed the systemic immune response within 28 days of injury and found no evidence that the acute inflammatory response is associated with subsequent CRPS development.

Our findings appear contrary to the prevailing theory for the development of CRPS [4, 45], and do not support the hypothesis that an exaggerated, systemic inflammatory response to the inciting injury is associated with the outcome. However, our methods do not exclude the possibility that an aberrant inflammatory response to the inciting event plays a role in the development of CRPS. A more localized inflammatory response at the site of the injury [46], or the duration, rather than the intensity of the immune response to injury, may be associated with the development of CRPS. It is possible that other factors, such as those in the central nervous system or behavioral domains, play a larger role in the development of CRPS. Although established CRPS has been associated with immune, central, and behavioral changes, it is not known whether these mechanisms drive the development of CRPS. In the present study we found that although individuals who developed CRPS did not express higher levels of systemic cytokines soon after their injury, they did experience more pain and psychological distress. This suggests that psychological and non-immune mechanisms of pain may play more of a role in CRPS development than an aberrant immune response [47].

We recognize limitations of the present study. First, we tested systemic cytokines as the main exposure. We may have obtained additional information about immune function by using complementary multi-omics approaches. However, we believe that the prevailing immune hypothesis necessitates a large, robust, and detectable shift in systemically expressed cytokines, rather than a subtle immune response only locally detected. Second, even though we recruited a large consecutive clinical fracture cohort and achieved a follow-up rate of 97.2%, the incidence proportion of CRPS in the recruited cohort (2.2%) was lower than expected, which reduced the precision of estimates of association. Our expectation was based on recent prospective studies that involved similar cohorts and used comparable diagnostic criteria to identify CRPS and reported incidence proportions of 4–7% [1, 2]. There are several possibilities for our low incidence proportion. Our cohort was younger (mean age of 39 years versus 43–62 years) and had proportionally fewer females (34% versus 51–83%) than the previously cited studies. This may be of importance because some studies have suggested that older women have a higher risk of developing CRPS [3, 48] The low incidence of CRPS may also reflect as yet unidentified clinical factors that minimize the development of CRPS. Because participants were recruited from specialized hand units that may have implemented early anti-inflammatory pharmacological therapies, it is conceivable that these strategies contributed to positive clinical outcomes and thus a lower incidence of CRPS. An alternate explanation is that the low CRPS incidence could reflect the diagnostic process in our study. However, we suspect this is unlikely as we applied a rigorous and sensitive standardized process to diagnose CRPS. We used a two-stage diagnostic process that began with a telephone screen. In order to maximize diagnostic sensitivity during the telephone call, we used multiple lay-language descriptors and examples for each CRPS sign and symptom (Appendix 1 in Additional file 2); we also performed in-person assessments on people who reported pain and at least two of the three signs required for a diagnosis of CRPS. As such, we do not believe that the low incidence diagnosis of CRPS reflects a misclassification of outcome but instead provides valuable epidemiological data. Finally, we did not make a distinction between CRPS I and II diagnostic subtypes. While we acknowledge that including individuals with CRPS arising from nerve injury may have introduced noise to the data, our aim was to identify whether a common immune response has a role in the development of CRPS [49].

The results of our secondary analyses were consistent with those of the primary analyses: they do not provide support for the idea that systemic inflammation plays a central role in the development of CRPS after a fracture. There is a caveat, however – there were wide confidence intervals around the estimated odds ratios. Therefore, we cannot definitively rule out an association between cytokine concentrations and the development of CRPS. None of the cytokines showed very large ORs (> 5) across the primary and secondary analyses (Fig. 2).

Because it is not known what threshold concentrations of the cytokines might affect the development of CRPS, we nominated that exposure to elevated cytokines occurred when cytokines exceeded the 80th centile for controls. To partially mitigate the risk of misclassification of exposure we also performed secondary analyses where cytokine concentrations were entered into a logistic model as continuous variables. The secondary analyses confirmed the primary analyses and did not provide any evidence to support the theory that elevated levels of cytokines were associated with CRPS. It is possible that other aspects of inflammation, such as local inflammation at the site of injury or the activation of central neuroinflammatory mechanisms are uniquely associated with the development of CRPS. While we would expect to see changes in systemically circulating cytokines when peripheral and central inflammatory mechanisms are activated, the precise relationships between local, central nervous system, and systemic concentrations of inflammatory mediators have not been elucidated in humans.

## Conclusion

In this case-control study nested in a prospective cohort study, there was no evidence that early post-injury expression of systemic cytokines was associated with a CRPS diagnosis 16 weeks after injury. This study does not provide support for the hypothesis that innate immune activation has a determinative role in the development of CRPS.

## Electronic supplementary material

Below is the link to the electronic supplementary material.

Additional file 1: STROBE checklist for case-control studies.

Additional file 2: Appendix 1: Telephone assessment form; Appendix 2: Objective assessment form; Supplementary Table 1. Association between exposure and the risk of CRPS, treating cytokine levels as continuous; Supplementary Table 2. Association between cytokine exposure and the risk of (IASP) CRPS; Supplementary Table 3. Raw cytokine data.

## Data Availability

The datasets generated and/or analyzed during the current study are not publicly available due to ethical requirements but are available from the corresponding author on reasonable request.

## List of abbreviations

CRPS: complex regional pain syndrome.
HLA: human leukocyte antigen.
IQR: interquartile range.
CI: confidence interval.
NRS: numerical rating scale.
DASS: Depression, Anxiety and Stress Scale.
quickDASH: Disabilities of the Arm, Shoulder and Hand short version.
CV: coefficient of variation.
LLOQ: lower limit of quantification.
ULOQ: upper limit of quantification.
RR: relative risk.
OR: odds ratio.
