# Supplementary material for: Post-fracture serum cytokine levels are not associated with a later diagnosis of complex regional pain syndrome: a case-control study nested in a prospective cohort study
Source: BMC Neurol. 2022 Oct 12;22:385. doi: 10.1186/s12883-022-02910-z (PMC9555076; doi:10.1186/s12883-022-02910-z)
Supplement: Supplementary file 2 — Additional file 2: Appendix 1: Telephone assessment form; Appendix 2: Objective assessment form; Supplementary Table 1. Association between exposure and the risk of CRPS, treating cytokine levels as continuous; Supplementary Table 2. Association between cytokine exposure and the risk of (IASP) CRPS; Supplementary Table 3. Raw cytokine data. [file 12883_2022_2910_MOESM2_ESM.docx]

**Additional file 2**

Parkitny et al. Post-fracture serum cytokine levels are not associated with a later diagnosis of Complex Regional Pain Syndrome: a case-control study nested in a prospective cohort study

Appendix 1: Telephone assessment form

Appendix 2: Objective assessment form

Supplementary table 1. Association between exposure and the risk of CRPS, treating cytokine levels as continuous

Supplementary table 2. Association between cytokine exposure and the risk of (IASP) CRPS

Supplementary table 3. Raw cytokine data

**Appendix 1: Telephone assessment form**

**Appendix 2: Objective assessment form**

**Supplementary table 1. Association between exposure and the risk of CRPS, treating cytokine levels as continuous**

| Cytokine | CRPS Diagnosis OR | 95% CI lower | 95% CI upper | p |
| --- | --- | --- | --- | --- |
| IL-1β | 0.82 | 0.35 | 1.92 | 0.65 |
| IL-10 | 0.35 | 0.05 | 2.27 | 0.27 |
| IFN-α | 0.68 | 0.11 | 4.42 | 0.69 |
| IL-6 | 0.90 | 0.26 | 3.06 | 0.86 |
| IL-12 | 0.44 | 0.04 | 4.65 | 0.50 |
| RANTES | 1.15 | 0.23 | 5.67 | 0.86 |
| eotaxin-1 | 1.80 | 0.19 | 16.76 | 0.61 |
| IL-13 | 0.77 | 0.08 | 7.45 | 0.82 |
| IL-15 | 1.11 | 0.42 | 2.97 | 0.83 |
| IL-17 | 0.48 | 0.13 | 1.82 | 0.28 |
| MIP-1α | 0.96 | 0.37 | 2.54 | 0.94 |
| GM-CSF | 1.01 | 0.40 | 2.58 | 0.98 |
| MIP-1β | 0.54 | 0.12 | 2.37 | 0.41 |
| MCP-1 | 0.62 | 0.05 | 6.91 | 0.70 |
| IL-5 | 0.52 | 0.18 | 1.46 | 0.21 |
| IFN-γ | 0.56 | 0.14 | 2.17 | 0.40 |
| TNF-α | 0.84 | 0.26 | 2.70 | 0.78 |
| IL-1Ra | 0.18 | 0.04 | 0.86 | 0.03 |
| IL-2 | 0.97 | 0.31 | 2.98 | 0.95 |
| IL-7 | 1.27 | 0.48 | 3.38 | 0.63 |
| IP-10 | 0.76 | 0.10 | 5.69 | 0.79 |
| IL-2r | 0.47 | 0.09 | 2.33 | 0.36 |
| MIG | 1.28 | 0.58 | 2.82 | 0.55 |
| IL-4 | 0.66 | 0.25 | 1.76 | 0.41 |
| IL-8 | 0.42 | 0.09 | 1.93 | 0.26 |

Results of secondary outcome analysis showing estimated odds ratios for the association between (for each cytokine) exposure and the risk of CRPS, treating (log10) cytokine levels as continuous. Intra-assay coefficients of variation (CV) are provided for each cytokine. OR = Odds ratio; CI = confidence interval; IL-1β = interleukin-1 beta; IL-10 = interleukin-10; IFN-α = interferon alpha; IL-6 = interleukin-6; IL-12 = interleukin-12; RANTES = Regulated on Activation, Normal T Cell Expressed and Secreted; IL-13 = interleukin-13; IL-15 = interleukin-15; IL-17 = interleukin-17; MIP-1α = macrophage inflammatory protein-1 alpha; GM-CSF = granulocyte-macrophage colony stimulating-factor; MIP-1β = macrophage inflammatory protein-1 alpha; MCP-1 = monocyte chemoattractant protein-1; IL-5 = interleukin-5; IFN-γ = interferon gamma; TNF-α = tumor necrosis factor alpha; IL-1Ra = interleukin 1 receptor antagonist; IL-2 = interleukin-2; IL-7 = interleukin-7; IP-10 = interferon gamma-induced protein-10; IL-2r = interleukin-2 receptor; MIG = monokine induced by interferon-gamma; IL-4 = interleukin-4; IL-8 = interleukin-8.

**Supplementary table 2. Association between cytokine exposure and the risk of (IASP) CRPS**

| Cytokine | CRPS Diagnosis OR | 95% CI lower | 95% CI upper | p |
| --- | --- | --- | --- | --- |
| IL-1β | 0.52 | 0.20 | 1.40 | 0.20 |
| IL-10 | 0.96 | 0.31 | 2.94 | 0.94 |
| IFN-α | 0.80 | 0.33 | 1.92 | 0.62 |
| IL-6 | 1.12 | 0.49 | 2.56 | 0.78 |
| IL-12 | 0.86 | 0.37 | 2.00 | 0.72 |
| RANTES | 1.24 | 0.57 | 2.68 | 0.59 |
| eotaxin-1 | 0.73 | 0.30 | 1.74 | 0.47 |
| IL-13 | 2.24 | 0.96 | 5.26 | 0.06 |
| IL-15 | 1.05 | 0.45 | 2.41 | 0.92 |
| IL-17 | 1.70 | 0.17 | 16.47 | 0.65 |
| MIP-1α | 0.61 | 0.24 | 1.52 | 0.29 |
| GM-CSF | 2.49 | 0.73 | 8.49 | 0.15 |
| MIP-1β | 0.66 | 0.26 | 1.65 | 0.38 |
| MCP-1 | 0.74 | 0.31 | 1.76 | 0.49 |
| IL-5 | 0.91 | 0.11 | 7.95 | 0.94 |
| IFN-γ | 2.36 | 0.47 | 11.83 | 0.30 |
| TNF-α | 1.34 | 0.55 | 3.26 | 0.52 |
| IL-1Ra | 0.59 | 0.23 | 1.48 | 0.26 |
| IL-2 | 0.95 | 0.40 | 2.28 | 0.91 |
| IL-7 | 0.95 | 0.41 | 2.21 | 0.90 |
| IP-10 | 0.81 | 0.35 | 1.87 | 0.62 |
| IL-2r | 0.40 | 0.14 | 1.19 | 0.10 |
| MIG | 1.43 | 0.68 | 3.02 | 0.35 |
| IL-4 | 1.30 | 0.42 | 4.05 | 0.65 |
| IL-8 | 0.43 | 0.16 | 1.16 | 0.09 |

Results of secondary outcome analysis showing estimated odds ratios for the association between (for each cytokine) exposure and the risk of CRPS (IASP criteria). Analysis is adjusted for age (years), gender (male, female) and day since injury. Intra-assay coefficients of variation (CV) are provided for each cytokine. OR = Odds ratio; CI = confidence interval; IL-1β = interleukin-1 beta; IL-10 = interleukin-10; IFN-α = interferon alpha; IL-6 = interleukin-6; IL-12 = interleukin-12; RANTES = Regulated on Activation, Normal T Cell Expressed and Secreted; IL-13 = interleukin-13; IL-15 = interleukin-15; IL-17 = interleukin-17; MIP-1α = macrophage inflammatory protein-1 alpha; GM-CSF = granulocyte-macrophage colony stimulating-factor; MIP-1β = macrophage inflammatory protein-1 alpha; MCP-1 = monocyte chemoattractant protein-1; IL-5 = interleukin-5; IFN-γ = interferon gamma; TNF-α = tumor necrosis factor alpha; IL-1Ra = interleukin 1 receptor antagonist; IL-2 = interleukin-2; IL-7 = interleukin-7; IP-10 = interferon gamma-induced protein-10; IL-2r = interleukin-2 receptor; MIG = monokine induced by interferon-gamma; IL-4 = interleukin-4; IL-8 = interleukin-8.

**Supplementary table 3. Raw cytokine data**

|  | Cases in-range | |  |
| --- | --- | --- | --- |
| Cytokine | N | Percent | LLOD |
| IL-1β | 191 | 48.10% | 4.3 |
| IL-10 | 199 | 50.10% | 10 |
| IFN-α | 395 | 99.50% | 8.2 |
| IL-6 | 250 | 63.00% | 2 |
| IL-12 | 394 | 99.20% | 5.1 |
| RANTES | 317 | 79.80% | 7.5 |
| eotaxin-1 | 389 | 98.00% | 2.1 |
| IL-13 | 53 | 13.40% | 14.1 |
| IL-15 | 330 | 83.10% | 14 |
| IL-17 | 70 | 17.60% | 9 |
| MIP-1α | 319 | 80.40% | 8 |
| GM-CSF | 181 | 45.60% | 8.1 |
| MIP-1β | 397 | 100.00% | 4.5 |
| MCP-1 | 396 | 99.70% | 6.6 |
| IL-5 | 154 | 38.80% | 2 |
| IFN-γ | 101 | 25.40% | 4 |
| TNF-α | 286 | 72.00% | 3 |
| IL-1Ra | 395 | 99.50% | 14.9 |
| IL-2 | 152 | 38.30% | 5.1 |
| IL-7 | 170 | 42.80% | 8.3 |
| IP-10 | 394 | 99.20% | 2.2 |
| IL-2r | 392 | 98.70% | 11.3 |
| MIG | 258 | 65.00% | 2.5 |
| IL-4 | 138 | 34.80% | 20.3 |
| IL-8 | 388 | 97.70% | 5.1 |

LLOD = lower limit of detection; IL-1β = interleukin-1 beta; IL-10 = interleukin-10; IFN-α = interferon alpha; IL-6 = interleukin-6; IL-12 = interleukin-12; RANTES = Regulated on Activation, Normal T Cell Expressed and Secreted; IL-13 = interleukin-13; IL-15 = interleukin-15; IL-17 = interleukin-17; MIP-1α = macrophage inflammatory protein-1 alpha; GM-CSF = granulocyte-macrophage colony stimulating-factor; MIP-1β = macrophage inflammatory protein-1 alpha; MCP-1 = monocyte chemoattractant protein-1; IL-5 = interleukin-5; IFN-γ = interferon gamma; TNF-α = tumor necrosis factor alpha; IL-1Ra = interleukin 1 receptor antagonist; IL-2 = interleukin-2; IL-7 = interleukin-7; IP-10 = interferon gamma-induced protein-10; IL-2r = interleukin-2 receptor; MIG = monokine induced by interferon-gamma; IL-4 = interleukin-4; IL-8 = interleukin-8.
